# Genome wide SNP discovery, analysis and evaluation in mallard (*Anas platyrhynchos*)

**DOI:** 10.1186/1471-2164-12-150

**Published:** 2011-03-16

**Authors:** Robert HS Kraus, Hindrik HD Kerstens, Pim Van Hooft, Richard PMA Crooijmans, Jan J Van Der Poel, Johan Elmberg, Alain Vignal, Yinhua Huang, Ning Li, Herbert HT Prins, Martien AM Groenen

**Affiliations:** 1 Resource Ecology Group, Wageningen University, P.O. Box 47, 6700 AA, Wageningen, The Netherlands; 2 Animal Breeding and Genomics Centre, Wageningen University, Marijkeweg 40, Wageningen, 6709 PG, the Netherlands; 3 Aquatic Biology and Chemistry, Kristianstad University, SE-291 88, Kristianstad, Sweden; 4 UMR Génétique Cellulaire, Centre INRA de Toulouse, 31326 Castanet-Tolosan France; 5 State Key Laboratory for Agrobiotechnology, China Agricultural University, Beijing 100094, PR China

## Abstract

**Background:**

Next generation sequencing technologies allow to obtain at low cost the genomic sequence information that currently lacks for most economically and ecologically important organisms. For the mallard duck genomic data is limited. The mallard is, besides a species of large agricultural and societal importance, also the focal species when it comes to long distance dispersal of Avian Influenza. For large scale identification of SNPs we performed Illumina sequencing of wild mallard DNA and compared our data with ongoing genome and EST sequencing of domesticated conspecifics. This is the first study of its kind for waterfowl.

**Results:**

More than one billion base pairs of sequence information were generated resulting in a 16× coverage of a reduced representation library of the mallard genome. Sequence reads were aligned to a draft domesticated duck reference genome and allowed for the detection of over 122,000 SNPs within our mallard sequence dataset. In addition, almost 62,000 nucleotide positions on the domesticated duck reference showed a different nucleotide compared to wild mallard. Approximately 20,000 SNPs identified within our data were shared with SNPs identified in the sequenced domestic duck or in EST sequencing projects. The shared SNPs were considered to be highly reliable and were used to benchmark non-shared SNPs for quality. Genotyping of a representative sample of 364 SNPs resulted in a SNP conversion rate of 99.7%. The correlation of the minor allele count and observed minor allele frequency in the SNP discovery pool was 0.72.

**Conclusion:**

We identified almost 150,000 SNPs in wild mallards that will likely yield good results in genotyping. Of these, ~101,000 SNPs were detected within our wild mallard sequences and ~49,000 were detected between wild and domesticated duck data. In the ~101,000 SNPs we found a subset of ~20,000 SNPs shared between wild mallards and the sequenced domesticated duck suggesting a low genetic divergence. Comparison of quality metrics between the total SNP set (122,000 + 62,000 = 184,000 SNPs) and the validated subset shows similar characteristics for both sets. This indicates that we have detected a large amount (~150,000) of accurately inferred mallard SNPs, which will benefit bird evolutionary studies, ecological studies (e.g. disentangling migratory connectivity) and industrial breeding programs.

## Background

The mallard (*Anas platyrhynchos*) is the world's most abundant and well-studied waterfowl species. Besides being an important game and agricultural species, it is also a flagship species in wetland conservation and restoration. Waterfowl (Anseriformes: Anatidae) and especially ducks are focal organisms in long distance dispersal of Avian Influenza in the wild [1-4], and the mallard has been identified as the most likely species to transport this virus [5,6].

As a general pattern, mallards breeding in temperate areas migrate from northern breeding grounds to more southerly wintering areas avoiding freezing conditions at breeding sites [7]. However, there are also non-migratory populations in Europe and elsewhere. Although some geographical patterns can be discerned from ringing recoveries on national levels, there is in Europe no clear delineation of flyways, and only little knowledge about the overall population structure from a genetic perspective [8]. This is exactly the situation for which Wink [9] proposed the use of SNPs to study bird migration in a population genetic framework. Since the number of SNPs necessary to detect low levels of differentiation is expected to be high (> 80) for highly mobile organisms [10,11], we aimed at a high throughput discovery of SNPs in the mallard. Large scale discovery of SNPs in the genome of the wild mallard might also provide a useful set of markers in the descendant, closely related domesticated duck (*Anas platyrhynchos domestica*). Being the third most consumed species on the poultry market globally [12], the domestic duck provides a valuable subject for detailed genomic studies. Nevertheless, genomic information about the domestic duck is limited to a few studies providing only low resolution linkage and physical maps [13,14]. Therefore our study also set out to facilitate duck breeding objectives by providing sufficient markers for improving the duck linkage map and allowing QTL mapping using SNPs.

A general limitation in developing a SNP-set in non-model organisms has been the unavailability of extensive genomic sequence information from multiple individuals that represent a sufficient portion of the genetic variability of the population or species under study. However, the Illumina sequencing technology [15-17] coupled with the approach of generating a reduced representation library (RRL) [18] has proven an efficient approach in solving this problem in the turkey (*Meleagris gallopavo*) [19] and great tit (*Parus major*) [20]. Also in rainbow trout [21], pig [22,23] and cattle [24] next generation sequencing of RRLs has been effective in the identification of considerable numbers of SNPs.

Here, we describe the discovery of more than 180,000 novel SNPs in the genome of the mallard, which currently lacks a published sequenced genome. Lacking this reference genome we initially aimed for paired-end sequencing on an Illumina Genome Analyzer of an RRL of fragments in the size range of 110-130 base pairs (bp) and with a read length of 76 bp. This would create an overlap between the forward and reverse DNA sequence reads of continuous sequences, permitting the reads to be merged. This in turn helps in providing sufficient flanking sequence (i.e. DNA sequence on either side) of a SNP which is a requirement for genotyping and is hard to retrieve in the absence of a reference genome. However, at the time when our study had started, genome sequencing of the domestic duck genome and *de novo *assembly was in progress and almost completed by the Beijing Genome Institute (BGI). This allowed for SNP discovery by next generation sequencing of an RRL of pooled wild mallard samples and mapping locations of almost 13 million of the resulting reads to a draft mallard reference sequence. Identified SNPs were compared with those observed within the reference genome sequence of domestic duck (Huang et al., in prep.) and EST sequencing (expressed sequence tags; Alain Vignal, unpublished data) resulting in more than 20,000 shared high quality SNPs. A set of putative SNPs can contain large numbers of incorrectly inferred SNPs (i.e. false positives) and thus we also aimed to estimate the quality of our set. Quality, here, is a measure of the reliability of the SNP set. This includes not only the percentage of false SNP inferences but also evaluation of the way in which these SNPs will be usable for many purposes; i.e., if they cover a large spectrum of minor allele frequencies, or if these were reliably inferred by our analyses (correlation between true allele frequencies and estimated allele counts, see below).

## Results

### Complexity reduction

We targeted for a sequencing depth of about 40 times at limited sequencing cost by sequencing a fraction, representing 5% of the mallard genome (reduced representation library (RRL) approach). Restriction enzymes were screened for suitability for RRL construction, with the goal of a 20-fold complexity reduction of the mallard genome within the targeted size range of 110-130 bp. Restriction enzyme analyses showed that these requirements are met by combining two RRLs, one created by enzymatic digestion with *Alu*I and one by digestion with *Hha*I, representing 4% and 1% of the mallard genome, respectively.

An *in silico *digest of the chicken genome, which is very similar [25,26], predicts similar genome fractions of the RRLs of 4.1% for *Alu*I, but only 0.2% for *Hha*I (data not shown). We prepared two pooled DNA samples of nine wild mallard individuals from three locations across Europe. To prepare the RRLs, we digested these samples with *Alu*I or *Hha*I and isolated fragments in the 110-130 bp size range from a preparative polyacrylamide gel. The genomic libraries were combined in the sequencing sample preparation procedure. Due to a lack of a reference genome we aimed for paired-end sequencing on an Illumina GAII of the combined RRLs and a sequence read length of 76 bases. This created an overlap between the forward and reverse reads of a pair which allows merging of the reads. Merging the reads helps in providing sufficient flanking sequence of a SNP. This sequence is necessary for genotyping and is hard to retrieve in the absence of a reference genome. Merged paired reads, possibly supplemented with single reads, are subsequently clustered for SNP discovery.

### Illumina sequencing and SNP detection

We generated 34.8 million 76 bp reads using three sequencing lanes on an Illumina GAII of which two lanes were run in paired-end mode. The raw data files from the sequencing instrument are deposited in the NCBI short read archive under accession number SRA024498. It was shown that a phred quality score [27] threshold of 12 ensures sufficient quality reads for SNP detection purposes [22,28]. Because the average base call quality score over all sequence reads dropped below 12 after read position 62, reads were trimmed to 62 bp. After trimming, we performed additional quality score based filtering (see methods) and finally we retained 16.6 million reads (47% of the raw data) of 62 bp length corresponding to a total of 1.03 billion bp of sequence information (Table 1). Of these reads 35% were single and 65% were paired reads. By creating RRLs 5% (69 Mb) of the mallard genome was represented (estimated size 1.38 billion bp, based on several entries in the Eukaryotic genome size databases [29]). From this we calculated that the raw sequencing data cover the sequence target 38 times (38×) whereas the quality filtered data provide a 16× target coverage. Using MAQ [30] 12,823,563 of the reads could be mapped onto the mallard reference genome (Huang et al., in prep.). A total of 632,163 putative SNPs were identified by MAQ [30] of which 122,413 candidate SNPs passed our applied SNP identification quality thresholds (see methods). This set of SNPs is further referred to as duck-RRL (d-RRL) and available in the dbSNP database under accession numbers ss263068950 - ss263191362.

**Table 1:** Summary of DNA sequence filtering results

	raw (76 bp)	l62 N. q12 o152^1^	%	paired-end	%	single	%
**reads**	34818352	16611852	47.7	10793170	65.0	5818682	35.0
**bases**	2547361732	1029934824	40.4	669176540	65.0	360758284	35.0

Paired and single sequence reads remaining after filtering raw reads.

^1^Raw sequences were filtered for length 62. Only reads without base-call errors (N or) were considered. Singly represented reads are required to have a per base-call quality of 12. Sequences more than four times overrepresented, based on the raw RRL coverage (38×, see methods) were discarded.

### SNP usability

**Figure 1:**
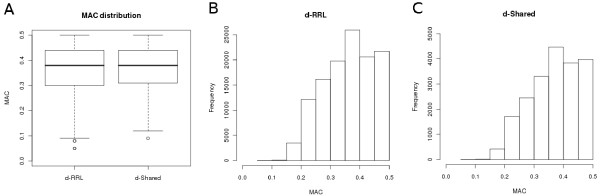
**Minor allele frequency distributions**. In boxplot A MAC distributions of d-RRL (SNPs identified in this study) and d-Shared (SNPs that d-RRL shares with d-EST or d-WGS (also see Venn diagram Figure 2D)) are compared. Histograms (B and C) show MAC distributions of d-RRL and d-Shared at a bin width of 0.05.

More than 98.8% of the SNPs were flanked by at least 40 bp on either side and met the requirements for probe design constraints for all genotyping platforms whereas all SNPs met the flanking sequence requirements for an iSelect (Illumina) genotyping assay. For the 2,565 SNPs that showed more than two alleles, we only considered the most frequently observed minor allele because tri- or tetra-allelic SNPs are very rare [31] and it is likely that most other minor alleles represented sequencing errors instead of true sequence variants. Analysis of the estimated allele counts of the SNPs in our dataset (Figures 1A and 1B) showed that we obtained a majority of SNPs with a high minor allele count (MAC, used here as a predictor of the minor allele frequency (MAF) of the real population data).

### SNP quality assessment

**Figure 2:**
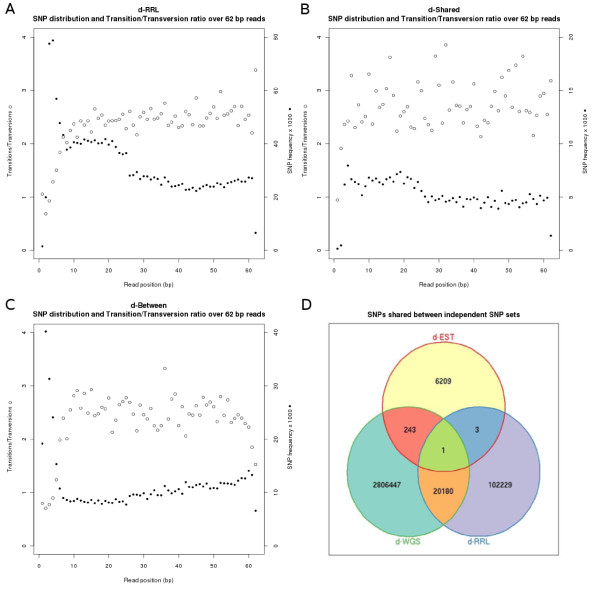
**SNP distributions within datasets and between datasets**. Diagrams A-C show the distribution of SNP predictions over the nucleotide position in the sequence reads for d-RRL, d-Shared and d-Between. Each filled dot represents the cumulative number of occurrences that the read position was involved in a SNP inference. Open dots represent the average TS:TV ratio of SNPs indentified in that read position. Diagram D shows how many SNPs are shared between independent SNP sets d-EST (SNPs identified by EST sequencing of domesticated duck (Vignal, unpublished data)), d-WGS (SNPs identified in the whole genome assembly of domesticated duck (Huang et al., in prep.)) and d-RRL (SNPs identified in RRL sequencing of wild mallard (this study)).

Sequencing errors are more abundant in the tails of next generation sequencing reads and are thought to cause an excess of false SNP predictions. An increase in the number of SNPs towards the end of the reads is expected if sequencing errors are the cause of a substantial number of predicted SNPs in the dataset. To validate our sequence filtering and SNP detection constraints we plotted the distribution of the SNPs over the 62 positions in the sequence reads (Figure 2A). Positions one, two and 62 all show an underrepresentation of SNPs whereas positions three, four and five show an overrepresentation. SNPs are equally distributed over read positions 6 to 25 and at 26 the number of SNPs per nucleotide position drops but after this remains more or less stable until position 62.

Because of the length of the RRL fragments (~110-130 bp), there is an overlap between paired forward and reverse reads (62 nucleotides each) from position 48 onwards. This overlap results in a higher sequence depth and a tiny increase in the number of SNPs being detected at these nucleotide positions (Figure 2A).

We estimated the possible errors in SNP calling due to sequencing errors by looking at transition (TS) - C/T pyrimidine to pyrimidine or A/G purine to purine changes - versus transversion (TV) ratios, which are all the four other possible pairs of changes. Random mutations or sequence differences due to errors should give a TS:TV ratio of 1:2. In reality, a bias due to a higher rate of C => T mutations due to the deamination of methylcytosines in CpG dinucleotides induce a much higher TS rate [32-35]. For instance in chicken, the TS:TV ratio is 2.2:1, based on the analysis of more than 3 million SNPs in the dbSNP database [36]. Our results show that the number of A/G substitutions almost equalled the number of C/T substitutions in the transitions class. Also the substitutions within the transversions class occurred in comparable frequencies (Table 2). The TS:TV ratio for d-RRL was 2.3:1 which is very similar to the 2.2:1 ratio found in chicken.

**Table 2:** Transition/transversion ratios in SNP subsets

	*Transitions*		*Transversions*				*Total*	*TS:TV^1^*
***subset***	*R*	*Y*	*M*	*W*	*S*	*K*		
**d-RRL**	42313	42602	9658	9051	9114	9675	122,413	2.3
**d-Shared**	7300	7442	1396	1227	1334	1484	20,184	2.7
**d-Between**	20156	21333	5464	5165	4804	4830	61,752	2.0

^1 ^= The transitions total divided by the transversions total for a data subset.

The two transitions and four transversions are abbreviated by their nucleotide ambiguity codes R, Y and M, W, S, K.

Sequencing errors were also evaluated per read position by plotting the TS:TV ratio observed over the 62 positions in the sequence reads (Figure 2). We observed steady expected TS:TV ratios for positions 7-61 whereas TS:TV ratios for positions 1-6 were lower and the TS:TV ratios for position 62 was higher than expected.

### SNP benchmarking

The *de novo *assembly of the domestic duck genome by the Beijing Genome Institute (BGI), covering both chromosomes of a single individual, resulted in the identification of 2,826,871 putative SNPs (further referred to as d-WGS; Huang et al., in prep.). Domestic duck EST sequencing identified a total of 6,456 SNPs (further referred to as d-EST) in protein coding regions of the genome (Alain Vignal, unpublished data).

To benchmark d-RRL we compared it with these two external and independent datasets and identified SNPs that are shared with either d-WGS or d-EST. We observed 20,180 SNPs (16.5%) in common between d-RRL and SNPs in the d-WGS dataset. Furthermore d-RRL had four SNPs in common with d-EST whereas d-WGS shared 244 SNPs with d-EST (Figure 2D). Only a single SNP was shared between all three datasets. The subset of SNPs (n = 20,184) that d-RRL shared with either of the two other SNP resources is further referred to as d-Shared. We analysed d-Shared by calculating the MACs and the TS:TV ratios (Figure 1C and Table 2). Furthermore, we plotted the TS:TV ratio per read position and the distribution of the SNPs over the 62 nucleotides of the sequence reads in the same way as was done for d-RRL. In d-Shared we observed a similar distribution of MACs compared to d-RRL (Figure 1C). The distribution of the SNPs in d-Shared detected on read positions 7-62 is similar to that observed for d-RRL; however, d-Shared shows a higher variation in the amount of SNPs between the read positions (Figure 2B). Also, TS:TV ratios at these read positions were similar with slightly more variation per read position in d-Shared.

Although reduced, also d-Shared showed a peak of the SNP distribution on read positions three to six, as we observed in d-RRL. However, TS:TV ratios for these positions were at the expected level of >2.3 indicating that most SNPs in these read positions likely resulted from true nucleotide polymorphisms. Finally, compared to d-RRL, the d-Shared subset of SNPs showed a higher average TS:TV ratio of 2.7 and indicated a relative increase of (C/T) over (A/G) transitions (Table 2).

### Domesticated versus wild mallard

Besides the identification of SNPs in wild mallards we also searched for nucleotide positions in the genome that show differences between the wild mallard population and the domesticated duck reference. We investigated nucleotides that where monomorphic within the wild mallard RRL consensus sequence data set but that differ from the corresponding non-polymorphic position in the domesticated duck reference. We identified 61,752 such SNPs (further referred to as d-Between) and assessed the quality of this set of SNPs by plotting the TS:TV ratio per nucleotide position and plotting the distribution of the SNPs over the 62 nucleotide positions in the sequence reads (Figure 2C). The distribution of SNPs predicted in the first six read positions showed a high peak whereas from position six to 62 the number of SNPs per read position was more or less constant, only slightly increasing towards the end. The TS:TV ratios were as expected except on the first six read positions and the end, where it was lower than expected. Compared to d-RRL and d-Shared the overall TS:TV ratio of d-Between was lower, 2:1, and showed a relative increase of (C/T) over (A/G) transitions (Table 2).

### The distribution of SNPs over the genome

**Figure 3:**
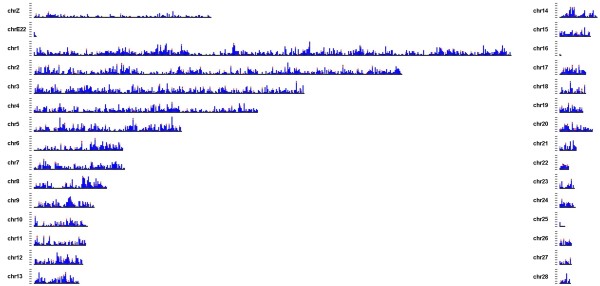
**Distribution of mallard SNPs uniquely mapped on the chicken genome**. In blue are 4272 mallard SNPs with a unique mapping position to the chicken genome (see text for mapping algorithms). 384 mapped SNPs that were selected for genotyping are in red. On the X-axis, the chicken genome in 400 kb intervals, and on the Y-axis, the frequency (0-15) of mapped mallard SNPs for a specific chicken genome interval is given.

Knowing genomic positions of SNPs as genetic markers is important. Many population genetic and genetic mapping applications rely on unlinked markers. Thus, for future use in generating a mallard linkage map and performing QTL studies in domestic and wild mallard it is essential that the SNPs are widely distributed over the genome. The domestic duck genome assembly that we used as a genome reference consists of thousands of scaffolds and contigs which are not assigned to chromosomes. Estimating the distribution of SNPs across this duck genome is therefore not possible using this sequenced reference. Consequently, the closest related available genome sequence (*Gallus gallus*, chicken; divergence time 80-90 million years ago, see discussion section) was used for estimating the physical distribution of the identified SNPs. Common and high quality mallard SNPs (d-Shared) were aligned to the chicken genome and the distribution of this SNP-set was plotted over the chicken chromosomes (Figure 3). A total of 4,272 SNPs could be mapped to unique locations evenly distributed over the chicken genome.

### SNP validation by genotyping

**Figure 4:**
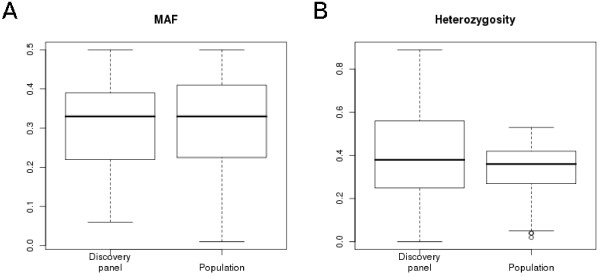
**Genotyping minor allele frequency and heterozygosity distributions**. Validation of the d-Shared subset involved genotyping of 384 selected SNPs on 765 ducks including the nine mallards that made up the SNP discovery panel. Minor allele frequency (MAF) and heterozygosity of SNPs were calculated for the discovery panel, as well as for the whole set of genotyped ducks.

The d-Shared subset of SNPs was validated by genotyping an animal panel consisting of 765 mallards using 384 predicted SNPs distributed uniformly over the chicken genome (Figure 3). A total of 364 (95%) SNPs gave reliable genotypes in the assay, and 363 (99.7%) of these were indeed proven to be polymorphic. The average minor allele frequency (MAF) was 0.32 in the animals that made up the discovery panel and 0.31 in the whole animal panel (Figure 4). The average heterozygosity was 0.39 in the discovery panel and 0.34 in the whole animal panel. The allele frequencies of polymorphic genotyped SNPs in the discovery pool showed a correlation of 0.72 with those derived from the sequence data in the discovery pool of nine animals.

## Discussion

This SNP study is the first large sequence variant discovery performed in mallards, as well as in any of the waterfowl. The availability of a large number of detected SNPs provides sufficient markers to study mallard population structure and migration in a population genetic framework. This large number of accurately inferred SNPs will also facilitate improved linkage maps of the mallard genome [13,14] and provide a sufficiently dense marker map to allow high resolution QTL studies in the domestic duck, further facilitating duck breeding. Furthermore, such high density linkage maps are essential for chromosomal assignment of the sequence scaffolds of the sequenced reference genome.

### SNP detection within a pool of wild European mallards

Initially, our study was designed to detect SNPs within a pool of wild European mallards by single-end and paired-end sequencing of a small fragment RRL. We targeted for genome libraries of sufficiently small fragments for paired reads to overlap. This allows the reads to be merged resulting in the complete sequence of the majority of the fragments in the RRL. Merged paired reads subsequently would serve as a reference genome. However, with the recent availability of a next generation sequenced domestic duck genome assembly, a reference based mapping approach became feasible, enabling a more efficient SNP identification approach. Our study shows that the overlap in generally lower-quality ends of paired-end sequence reads is beneficial in reference based SNP detection. An observed drop in the number of predicted SNPs after position 25 (Figure 2A) is explained by a drop in phred scores of the raw sequence data at exactly that position (data not shown). Subsequent filtering for quality scores eliminates more putative SNPs after read position 25. However, accounting for this inherent quality issue in the raw data, we observed that the number of SNPs being predicted per read position shows a tiny increase in the overlapping ends of our mate pairs whereas earlier studies [19,20,22] reported decreasing numbers of predicted SNPs per nucleotide position towards the end of sequence reads. The deamination of methylcytosines results in a thymine base. This reaction is especially frequent in CpG dinucleotides motifs, causing a much higher mutation rate from C to T than any other mutation type. As a consequence, TS:TV rations are much higher than expected, as for instance in chicken where it is 2.2:1 instead of the 1:2 ratio expected if mutations were random. A similar 1:2 for TS:TV ratio would be found in sequences if base differences were due to sequencing errors rather than true polymorphism (same as above). The TS:TV ratio of SNPs we predicted in the overlapping ends of our sequences remains in the expected range (Figure 2A) suggesting that these SNPs reflect true nucleotide polymorphisms. A local decrease in TS:TV ratio would be observed if SNPs in read positions (51-61 in d-RRL and 52-60 in d-Between) were caused by randomly introduced polymorphisms (e.g., sequencing errors). Thus we expect that the predicted SNPs represent true nucleotide polymorphisms. The increased number of SNPs at the overlapping ends can be explained by local higher sequence coverage, caused by sequence overlap of paired reads, resulting in a higher representation of DNA sequence variants. A higher coverage allows for multiple observations of the variant in low quality sequences, allowing it to pass MAQ's quality thresholds to call it a true SNP [30]. As a result, even more of the rare sequence variants in these overlaps will meet the minor allele occurrence constraint in our SNP detection method. An indication that the additionally identified SNPs at the read ends involve rare sequence variants is the lower representation of these SNPs in d-Shared.

### Ascertainment bias due to limited sequence depth

Besides limited sequencing depth also sequence quality is a limiting factor for inferring SNPs. This is illustrated by the overall trend in the number of predicted SNPs per read position in d-RRL and d-Shared (Figure 2A and 2B), which mirror the decreasing trend of average base call score per nucleotide position inherently present in Illumina sequencing (as also observed in our data set, data not shown). A similar trend is not observed in d-Between because here the SNPs are predicted from differences between the reference and the discovery panel of wild mallards. Read depth is less limiting in d-Between because it is only used to provide one unambiguous (consensus) base, deviating from the reference, of sufficient quality whereas in d-RRL the read-depth has to provide sufficient base calls for both the major allele and the minor allele to be considered a SNP.

Besides the unequal distribution of identified SNPs over the read positions also the underrepresentation of SNPs with a MAC <0.2 is an indicator of a coverage limitation. Due to the limited coverage, only SNPs that are present in multiple individuals in the discovery panel have a reasonable probability to meet the minor allele representation constraint set by our SNP detection method. More common alleles will pass the representation constraint more frequently than rare alleles resulting in an overrepresentation of common alleles and an underrepresentation of rare alleles.

### SNP set quality assessment by comparison

We identified a large number of putative SNPs in the sequenced mallard discovery panel by sampling ~5% of the mallard genome. Extrapolating the total number (d-RRL + d-Between) of identified SNPs would result in a SNP every ~375 bp. The actual number of true SNPs in the sets d-RRL and d-Between is expected to be lower considering the overrepresentation of predicted SNPs in the read positions one to six together with low TS:TV ratios in these read positions. Also the comparison of d-RRL with d-WGS, in which common true variants remained and false SNPs were discarded, show that SNPs predicted in read positions one to six should be used cautiously. The distribution of d-Shared does not show overrepresentation of SNPs on position one to six. Furthermore, expected TS:TV ratios in d-Shared were observed for positions three to six and expectedly lower TS:TV ratios in position one and two due to the RRL enzyme restriction motif. Therefore we think that a considerable fraction of SNPs in read positions one to six in d-RRL and d-Between are false positives. Because standard sequencing error rates of the Illumina GAII are low (< 0.5%) in the first 20 bases of a read [37] we expect that the first six bases in our sequence dataset were affected by non-standard, systematic, sequencing errors. These are most likely resulting from a combination of inadequate separation of sequencing clusters due to the restriction tag in the RRL and an overloaded sequencing flow cell (Kees-Jan Françoijs personal communication). This hypothesis is supported by the fact that quality scores were considered by the SNP inferring algorithm [30] and that two observations of the minor allele were required for a putative SNP making it unlikely that these numbers of false positives are due to standard sequencing errors. Low TS:TV ratios for SNPs at read position 61 and 62 in d-Between suggest that the SNPs from these positions should also be omitted. Subtracting SNPs from positions one to six (and position 61 and 62 in d-Between) results in 101,095 SNPs in d-RRL and 48,592 SNPs in d-Between that will likely yield good success rates in genotyping.

### Shared SNPs

We showed that d-RRL shares one sixth of the SNPs with d-WGS and an almost negligible number of SNPs with d-EST. ESTs only represent a few percent of the genome, of which only a fraction was sampled by the RRL. Due to this limited shared genome fraction and because SNPs in protein coding regions are rarer than in non-coding regions, a large overlap in SNPs between these sources was not expected. Between d-WGS and d-EST we observed a 2.6 times larger overlap, which can be explained by a more or less complete overlap in sampled genome fraction and a better representation of rare alleles in d-WGS. The relatively large overlap between d-WGS and d-RRL indicates a low genetic divergence between wild mallard and domestic duck. A relatively large fraction of shared SNPs between two independent studies also suggests a low false discovery rate. As stated earlier, the SNPs identified in this study will be used to study mallard population structure and movements in a population genetic framework [9]. Because the required number of genetic markers for such an analysis is small compared to the total amount of markers we generated [10], we selected SNPs from d-Shared that show an equal distribution over the chicken genome. This requirement greatly reduces the number of available markers since only a small fraction could be mapped (Figure 4) due to the relatively large evolutionary divergence time between chicken and ducks (80-90 million years ago, http://www.timetree.org)[38]. Genotyping of this SNP subset confirmed the expectation that SNPs that are shared between independent SNP detection studies yield a SNP set of high quality.

## Conclusions

When performing SNP identification studies using next generation sequence technologies, it is important to know what limitations in sensitivity and specificity can be expected, particularly at low sequence coverage. We show that sensitivity decreases with decreasing base calling quality towards the ends of sequence reads which can be compensated for by increasing the sequence coverage in the ends. SNP distribution and TS:TV ratio over read positions are helpful metrics for the assessment of systematic errors in the sequencing dataset in particular when statistics can be compared to a high quality subset of the data. We showed that the fairly large subset of predicted SNPs that is shared between independent SNP detection studies in wild and domestic duck is likely to represent true SNPs, and suggests a low divergence between these forms.

We present for the first time a solid and scalable genotyping environment applicable to mallards and its domestic form. Not only do we provide over 100,000 most reliable SNP markers that can be used in duck breeding and molecular genetics, we also evaluate a sub-set of 384 SNPs for use in ecological genetics. The power of this set combined with relatively low genotyping costs through down-scaling of the marker set will allow long needed studies into the molecular ecology of mallards with regard to various relevant topics, including the study of genetic variation and genetic structure, resolution of unresolved ambiguities of mallard migration systems or inference of both small and large scale movement patterns.

## Methods

### Sample collection and preparation

Mallard DNA samples were prepared from ethanol preserved whole blood collected from nine individuals from three locations across Europe: two females and a male each from Coto de Doñana (Spain), Northern Netherlands and Ottenby (Sweden). Each of these individuals was either directly caught from the wild, or was a first generation descendant from local wild mallard parents. Ducks were sampled under the approval of the animal ethical committee of Wageningen University; the Spanish Ministry of Environment and Consejeria de Medio Ambiente of Junta de Andalucia; the KNAW (Royal Dutch Academy of Sciences) Animal Experiment Commission; and the Swedish Board of Agriculture and its Research Animal Ethics Committee. DNA extraction was performed using the Gentra Systems Puregene DNA purification Kit according to the manufacturer's instructions. Briefly, ~200 μl blood was digested with 9 μg Proteinase K (Sigma) in Cell Lysis Solution (Gentra Systems) at 55°C over night. Proteins were subsequently precipitated with Protein Precipitation Solution (Gentra Systems) and spun down. DNA from the supernatant was precipitated with isopropanol and washed twice with 70% ethanol. DNA quantity and purity were measured using the Nanodrop ND1000. Possible degradation was inspected on an agarose gel and only high quality DNA samples were used to prepare the DNA pool. Equal amounts of DNA from the nine mallards were combined into two pools of 25 μg each. Aliquots of 5 μg for each pool were digested with either *Alu*I or *Hha*I (10 units per reaction, Pharmacia). The digested pools in O'range loading dye (Fermentas) were size-fractionated on precast 10% polyacrylamide in 1×TBE with the Criterion™ Cell (BioRad). The gel was run 190 minutes at 100 volt and stained for 30 minutes in ethidium bromide solution. After staining, the target fragment size range between 110-130 bp was sliced out of the gel. The gel slice was sheared by nesting a 0.5 ml Eppendorf tube (with a hole in the bottom formed with a needle) containing the gel slice inside a 2 ml Eppendorf tube, and centrifuged at 14000 rpm for 2 minutes. The sheared gel pieces were covered with 300 μl DNA recovery buffer (8 mM Tris pH 8.0, 0.08 mM EDTA, 1.25 M ammonium acetate), vortexed, and eluted at 4°C overnight, followed by 15 minutes incubation at 65°C. The slurry was divided over two Montage DNA gel extraction devices (Millipore) and centrifuged at 5000 g for 10 minutes to purify the eluted gel. DNA was precipitated by adding 1/10 volume 3 M sodium acetate pH 5.2, 1 volume isopropanol and 1/500 volume glycogen, washed with ethanol and resuspended in DNA hydration solution (Gentra Systems). The genomic libraries were combined and prepared using the Illumina Sample Preparation kit [39] and sequenced for 76 cycles with the Illumina GAII, Illumina Inc., USA, with a paired end module attached.

### SNP detection

Prior to analysis we applied quality filters to the raw reads. Due to the use of restriction enzymes *Alu*I and *Hha*I for creating the genomic libraries we expect that the sequence reads start with a 'C'. Therefore, reads not starting with 'C' were discarded as unreliable or contamination. All reads of the sequencing dataset were trimmed from the position where the average quality score dropped below 12. Reads containing a base that was called with a quality lower than 12 were discarded unless an identical copy of the read occurred in the dataset, since it is unlikely that two fragments of such a long sequence of nucleotides are identical by chance. We removed reads that - based on the theoretical raw sequencing coverage of the RRL (38×) - were more than four times overrepresented to limit the number of sequences from repetitive regions in the dataset. This is to prevent the prediction of SNPs within multi-copy genes or other repetitive regions [19,20].

As reference we used a domestic duck genome sequenced by next generation technology by the Beijing Genome Institute (Huang et al. in prep.). MAQ [30] was employed to map the quality filtered reads to the domestic duck genome with default parameters. Putative SNPs were tagged if the reads involved were mapped unambiguously to the reference. We filtered the MAQ [30] SNP output according to several rules: minimal map quality per read: 10; minimal map quality of the best mapping read on a SNP position: 10; maximum read depth at the SNP position: four times the actual coverage after quality filtering; minimum consensus quality: 10 [22]. We required that the minor allele at a polymorphic position in the reference was observed at least two times.

### EST-mapping

We mapped d-EST SNPs on the genome reference to identify their genomic locations whereas SNPs in d-RRL and d-WGS were predicted on an identical genome reference coordinate system. Mallard SNPs (with on average 116 bp of flanking sequence) being predicted in EST sequences by the group of Alain Vignal (INRA France, unpublished data) were mapped to the reference genome using GMAP [40]. Results were filtered for SNPs that aligned with 96% sequence identity.

### Comparative mapping

To examine the distribution of SNPs over the genome, we comparatively mapped our predicted SNPs (including 100 bp flanking sequence at each side) to the repeat masked chicken genome (assembly WASHUC2). Mapping was performed using BLAT [41] with parameters -oneOff = 1 -minIdentity = 70.

### SNP validation by genotyping

SNPs were validated by genotyping an animal panel using the Illumina GoldenGate^® ^Genotyping assay on an Illumina^® ^BeadXpress with VeraCode™ technology. Selection criteria for the SNPs were based on the Illumina design score (above 0.8) and the assayed 384 SNPs should distribute evenly along the chicken genome to minimise the extent of linkage between neighbouring SNPs. Oligo-nucleotides were designed, synthesised, and assembled into oligo pooled assays (OPA) by Illumina Inc. The Illumina OPA file can be found as Additional file 1 "GS0011809-OPA.opa". The 384 SNPs were genotyped in 765 animals which included domesticated ducks from a French (7 individuals) and a Chinese (189 individuals) genetic mapping population, non-*Anas platyrhynchos *duck species specimens (36 individuals), ~500 wild mallards from Europe, North America and Asia and the nine mallards that made up the SNP discovery panel. Genotyping results were analysed in Genome Studio (Illumina). Using the cor-function in R [42] the Pearson correlation between allele frequency estimated by sequencing and genotyping was calculated over 361 SNP loci that were polymorphic in the discovery panel genotyping by randomly selecting the major or minor allele.

## Authors' contributions

HHDK and RHSK designed and carried out SNP detection and drafted the manuscript. RHSK collected, prepared and genotyped DNA samples. HHDK interpreted genotyping results. NL and YH provided the BGI reference genome sequence of the domestic duck including the BGI SNPs and analytical support. AV provided the EST SNPs. MAMG and RPMAC coordinated the research and helped in drafting and revising the manuscript. PvH, JJvdP, JE and HHTP contributed to study design. All authors read, edited and approved the final manuscript.

## Supplementary Material

Additional file 1 **Oligo pooled assay (OPA) data file**. This file was used in the genotyping method as indicated in the methods section to generate the raw data. The genotyping assay can be re-ordered from Illumina using this file. Format is plain text, comma separated. Click here for file
